# Meaningful and Lasting Change – Psychotherapy in the Light of Evolutionary Processes

**DOI:** 10.32872/cpe.9859

**Published:** 2022-09-30

**Authors:** Andrew T. Gloster, Elisa Haller

**Affiliations:** 1 Division of Clinical Psychology and Intervention Science, Faculty of Psychology, University of Basel, Basel, Switzerland

**Keywords:** psychotherapy, evolution, processes of change

## Abstract

- Psychotherapies can lead to meaningful and lasting change.
- Evolutionary theory is relevant for understanding psychotherapy.
- Process-based approaches to conceptualizing psychotherapy can help organize clinical knowledge.
- Process-based approaches may be more useful than competitions between psychotherapy schools.

All psychotherapies aim to exact change. This basic tenant holds true as much for therapies that explicitly work with clients to alter the way they behave as it does for psychotherapies that try to help clients accept what is, stop trying to change, and thus manage to adapt. This much, we believe, is agreeable to all clients, practitioners, and researchers.

All psychotherapies also aim to exact change that is useful in clients’ lives. Whereas one can argue about how to define benefit (e.g., symptom reduction, increase in wellbeing, social integration, behavioral performance, etc.), a plethora of empirical evidence across many types of psychotherapies demonstrates that psychotherapy “works” (e.g., Gloster et al., 2020; Hofmann et al., 2012). Absent such data, it would nevertheless be logical that, by and large, clients must benefit in some way, lest they would not come back and healthcare systems would not spend capital on and regulate access to psychotherapy without a return on investment.

Similarly, all psychotherapies aim to exact lasting change. That is, clients and psychotherapists are working to establish meaningful changes that last beyond the psychotherapy itself. Here, large differences exist across psychotherapies: some explicitly address maintenance and generalization, whereas others are silent as how to help achieved gains “stick”. Nevertheless, research shows that change for many can be maintained for years following treatment.

As such, we believe it is uncontroversial that the basic tenants of evolution can be brought to bear on all psychotherapies: variation (change), selection (utility), and retention (lasting change). Developments in evolutionary science demonstrate that evolutionary processes are not limited to genetics, that they include processes that shape behavior and symbolic language (the bread and butter of psychotherapy), and can play out in much faster time spans than previously believed (Wilson et al., 2014).

One such attempt to conceptualize and organize empirically verified change processes in psychotherapy around evolutionary concepts is the process-based approach to psychotherapy (Hayes et al., 2019; Hofmann et al., 2022). Although its implications are not yet established, the theoretical groundwork is now ready to guide the next steps of empirical examination of candidate processes of change (Hayes et al., 2022). We believe this type of thinking is more promising than our fields’ history of fighting about which psychotherapy is better. It is also closer to clinical reality of the multi-method and multi-dimensional approach of most clinicians. The upshot here is that with concerted effort, clinical wisdom could be organized around evolutionary concepts (e.g., “meaningful variation was achieved for this client using the empirically established procedure of X”, etc.). Furthermore, this perspective is egalitarian and open to all psychotherapies, theories, and even our field’s favorite animal, the dodo bird. It may take time before the field concludes that nothing in psychotherapy makes sense except in the light of evolution – to borrow a famous phrase – but such a step could be meaningful change in itself.
